# Early-life maternal deprivation affects the mother-offspring relationship in domestic pigs, as well as the neuroendocrine development and coping behavior of piglets

**DOI:** 10.3389/fnbeh.2022.980350

**Published:** 2022-10-06

**Authors:** Ulrike Gimsa, Roberto Brückmann, Armin Tuchscherer, Margret Tuchscherer, Ellen Kanitz

**Affiliations:** ^1^Psychophysiology Group, Institute of Behavioural Physiology, Research Institute for Farm Animal Biology (FBN), Dummerstorf, Germany; ^2^EUROIMMUN Medizinische Labordiagnostika AG, Lübeck, Germany; ^3^Service Group Statistical Consulting, Institute of Genetics and Biometry, Research Institute for Farm Animal Biology (FBN), Dummerstorf, Germany

**Keywords:** early-life stress, immunomodulation, neuroendocrine, programming effects, social support, swine

## Abstract

Early-life adversity may have programming effects on the psychological and physiological development of offspring. Domestic pigs (*Sus scrofa*) are an excellent model species for studying these effects because of their many physiological similarities to humans. Piglets from 10 sows were subjected to daily 2-h maternal deprivation on postnatal days (PND) 2–15 alone (DA) or in a group of littermates (DG). Control piglets (C) from 10 sows stayed with their mothers. Mother-offspring interaction, milk oxytocin, and cortisol were analyzed. An open-field/novel-object (OF/NO) test was performed with piglets on PNDs 16 and 40. Plasma cortisol and immune parameters were determined on PND 5 and 16. Two piglets from each group and sow were sacrificed on PND 20 and stress-related gene expression in the limbic system and prefrontal cortex (PFC), as well as splenic lymphocyte proliferative abilities, were examined. The milk cortisol of sows increased during the first separation of mother and offspring on the second day of lactation, whereas milk oxytocin did not change. The increase in cortisol by the OF/NO test on PND 16 was greater in C piglets than in DA and DG ones. DA piglets showed less agitated behavior than DG and C piglets in the OF/NO test at PND 16, but appeared more fearful. On PND 40, DA piglets showed more arousal than DG and C piglets in the OF/NO test. Neither plasma IgA nor N/L ratios in blood nor mitogen-induced proliferation of spleen lymphocytes were affected by deprivation. We found a higher mRNA expression of CRHR1 in the hypothalamus and a higher expression of MR in the hippocampus in DA piglets than in DG ones. The expression of GR, MR, and CRHR1 genes in the PFC was reduced by maternal deprivation, however, the expression of arginine vasopressin and oxytocin receptors was not affected. Repeated maternal deprivation induces sustained effects on stress reactivity and behavior of domestic piglets. Some of these effects were buffered by the presence of littermates. In addition, we found sex-specific differences in behavior and gene expression.

## Introduction

In mammals, the lactation period is a time during which many ontogenetic processes, such as brain maturation, immune system development, and mother-infant bonding, are still ongoing (Bailey et al., 2005; Mogi et al., 2011; Lessard et al., 2018; Tanabe and Yamashita, 2018; Zhang et al., 2019). Thus, it is a phase of increased vulnerability, and early-life adversity may have lasting effects on offspring development.

Stress during the lactation period of pigs, resulting in elevated cortisol concentrations in the blood and milk, can disrupt the sow-piglet relationship and lead to piglet losses (Edwards, 2002; Kirkden et al., 2013). Despite improved housing and management conditions, piglet losses are as high as 15%, due partly to stress during the first weeks of lactation (Hoy, 2004).

Disruptions in the sow-piglet relationship may result from psychological stress to piglets in the early-postnatal period of life. In laboratory animals and primates, psychosocial stress in the postnatal period leads to changes in stress adaptation and can have sustained adverse effects on immune responses (Lyons et al., 2000; Levine, 2001; Black, 2002). However, results from rodent models do not readily translate to pigs because newborn piglets do not have a stress-hyporesponsive phase like rodents (Kanitz et al., 1999) and differ from rodents in their postnatal brain development (Lind et al., 2007; Kanitz et al., 2011; Conrad and Johnson, 2015). Besides changes in parameters of the hypothalamic-pituitary-adrenal (HPA) axis, the neuropeptides vasopressin (AVP) and oxytocin (OXT) and their receptors in different brain areas, which are involved in social behavior, may be affected by maternal separation (Kompier et al., 2019).

Husbandry challenges in pigs have both physical and psychological components. These challenges include sows’ and littermates’ deprivation at weaning and mixing with unfamiliar conspecifics. These challenges may have acute and long-term effects on the immunocompetence of pigs (Gimsa et al., 2018). Even a single episode of maternal deprivation and social isolation alters ethological and physiological adaptive responses (Kanitz et al., 2009, 2014; Tuchscherer et al., 2009, 2014), sensitizes peripheral and central stress regulation in response to bacterial infection, and thereby increases disease severity (Tuchscherer et al., 2018). Research on the ability of conspecific social support to mitigate negative stress consequences and the mechanisms involved in them is still at an early stage (Kanitz et al., 2014, 2016; Tuchscherer et al., 2016). The question of whether exposure of piglets to psychosocial stress also causes stress to the sow has not been investigated so far.

We hypothesize that repeated maternal deprivation of piglets affects the mother-offspring relationship and stresses the sow. For piglets, we predict that the deprivation of mother and littermates in a group of familiar conspecifics is perceived as being less stressful than experiencing the same stressors alone. Piglets were separated from their mothers and littermates alone or with familiar conspecifics for 2 h daily over 2 weeks to test these hypotheses. During that time, maternal behavior, the neuroendocrine response of sows to separation, and sow-offspring interactions were observed. The plasma cortisol and immune parameters of piglets were also determined. One day and 25 days after the deprivation period, the piglets were behaviorally challenged by an open-field/novel-object (OF/NO) test to assess the effects of different psychosocial treatments on their behavioral reactivity in the short and long-term. Moreover, we investigated changes in gene expression in those brain areas involved in regulating the HPA axis.

## Materials and methods

### Animals and experimental design

All procedures involving animal handling and treatment were performed according to the German Animal protection law and were approved by the local authorities (Landesamt für Landwirtschaft, Lebensmittelsicherheit und Fischerei, Mecklenburg-Vorpommern, Germany; LALLF M-V/TSD/7221.3-1.1-003/18).

A total of 200 piglets from 20 sows of the German Landrace (*Sus scrofa*) in their second to fourth parity were born and raised in the experimental pig facility of the Research Institute for Farm Animal Biology (Dummerstorf, Germany). Immediately after birth, the litter size was standardized to 10 piglets per sow. Sows and piglets were housed in loose farrowing pens (6 m^2^) with a plastic floor covered with sawdust and a constantly heated area for laying down on (28 ± 1°C) for the piglets and unrestricted access to feed and water. The light regime was set to 12/12 h light/dark with lights on at 6:00 a.m. The piglets and sows were examined in 10 replicates, and in each replicate, two litters were randomly assigned to a deprivation and control litter. The two sows with litters were housed in the same farrowing unit in neighboring farrowing pens. They had acoustic and olfactory contact during the first 8 days, while restricted to the farrowing crate. Later, after release from the crates, they could have nose contact through the fence. Within each deprivation litter, half of the piglets were randomly assigned to each of the social stress procedures with a balanced sex ratio: (1) maternal and littermate deprivation, i.e., total social isolation (five piglets were separated alone, DA); and (2) maternal and partial littermate deprivation (five piglets were separated as a group, DG). On postnatal days (PND) 2–15, the piglets of both treatment groups were deprived for 2 h in the morning (7:00–9:00) in separate test rooms located within the same experimental station. During the social deprivation period, the piglets were placed in opaque boxes either alone (60 × 40 × 32 cm) or as a family group (159 × 68 × 56 cm) with sawdust on the floor and adequate air passage. The socially deprived piglets were kept under the same air and temperature conditions as in the farrowing pen. The control litter (C) piglets remained undisturbed in the farrowing pen during this time.

Milk samples were collected from sows on the second day of lactation before the piglets were separated (at 7:00) and immediately after the piglets had returned (at 10:00). On lactation days 9 and 15, milk samples were only collected immediately after the return of the piglets because taking milk before separation would have been an additional stress factor for the piglets. Blood samples of piglets were taken on PND 5 at 7:00 and PND 16 before (at 7:00) and immediately after the open-field/novel-object (OF/NO) test (8:00–12:00). The OF/NO test was repeated on PND 40. Two piglets (one male, one female) from each group and litter were sacrificed on PND 20 for brain tissue analysis.

Weaning of the piglets was performed after 28 days by transferring the deprivation and control litters to a common weaning pen with controlled light and temperature conditions and commercial feed from an automatic feeder. Feed and water were available to the animals *ad libitum* (for more details on husbandry conditions, see Brückmann et al., 2020).

### Behavioral observations

#### Mother-offspring interactions

Maternal behavior and sow-piglet interactions were determined by continuous, direct observation of piglets and sows over three successive suckling bouts. The observation started after the return of all piglets from deprivation (at 10:00) simultaneously for the control and deprivation litters.

Immediately before any suckling events, the occurrence of the following behaviors was counted, and relative frequencies were calculated based on all litters and suckling events. These were, (1) “grunt”: sow initiated suckling by emitting grunts to call the piglets; (2) “piglet calls”: piglets initiated suckling by calling for attention; (3) “pre-lying behavior”: sow exhibited pre-lying behavior, such as scratching with one front leg, sniffing the ground, counting/sorting piglets, nest-building behavior (digging in straw; looking for material); (4) “lying down”: sow shows controlled and slow bending of front and hind legs; turning to the side is also controlled and slow; (5) “piglet contact”: sow reacts to piglets near her head by contact and sniffing; (6) “response to human”: sow reacts very nervously or aggressively to people nearby; (7) “response to screams”: sow shows restless behavior at piglet screams and looks for them; and (8) “suckling intervals”: time interval between nutritive suckling bouts.

#### Open-field/novel-object test

A combined 10-min OF/NO test was used to investigate the influence of early-postnatal psychosocial stress on the behavioral reactivity of piglets. Testing took place in a separate, noise-attenuated room with a square test arena (2.80 × 2.80 × 1.25 m) and on two different days of life (PNDs 16, 40). Piglets were tested in random order regardless of sex. The piglets’ behaviors were recorded during the 10-min test period using the focal sampling method with Observer XT 13 software (Noldus Information Technology). The first 5 min represented the OF situation. Immediately afterward, a foreign object (PND 16: plastic shoe; PND 40: 1.5 kg medicine ball) was lowered from the ceiling and remained suspended approx. 10 cm above the floor in the arena until the end of the 10-min test period (NO situation). The arena was cleaned between the tested piglets. First, urine and feces were removed with dry cloths. Then the arena was washed thoroughly with soapy water. The OF/NO tests were always performed by the same persons. The behaviors listed below were recorded and analyzed in terms of duration, frequency, and latency. The observed behaviors were defined as (1) “standing”: no active locomotion, standing on at least three legs; (2) “lying”: the ground is touched with all four legs and the abdomen; (3) “locomotion”: active locomotion with at least with two steps; (4) “escape”: active attempt to leave the arena by jumping up the wall; (5) “defecation”: excretion of feces; (6) “urinating”: discharge of urine; (7) “object contact”: active touching or manipulation of the novel object with the snout; and (8) “vocalization”: active vocalizations (grunting, screaming) (Kanitz et al., 2004; Puppe et al., 2007).

#### Sampling of milk, blood, and tissue

#### Milk

Milk was collected by gentle, manual teat massage at the start of a suckling bout. The milk was centrifuged at 40,000 × g for 1 h at 4°C. The phase in the middle was centrifuged again at 20,000 × g for 30 min at 4°C. The supernatant was stored at –20°C until analysis.

#### Blood

Blood samples were taken while piglets were in a supine position by anterior vena cava puncture. The whole procedure lasted approx. 1 min. The samples were transferred to ice-cooled polypropylene tubes containing EDTA solution and placed on ice. A blood sample of 100 μl was stored separately for differential blood counts (see section “Determination of the neutrophil to lymphocyte ratio from blood”). The rest was centrifuged at 2,000 × g at 4°C for 15 min. The resulting plasma was stored at –20°C until analysis.

#### Tissue

On PND 20, one male and one female piglet from each group (DA, DG, C) and sow were anesthetized with Ursotamin^®^ (100 mg/mL ketamine hydrochloride, Serumwerk Bernburg AG, Bernburg, Germany) and Stresnil^^®^^ (40 mg/mL Azaperone, Elanco, Homburg, Germany) and euthanized by an intravenous injection of T61^^®^^ (embutramide/mebezonium iodide/tetracaine hydrochloride, Intervet, Unterschleiβheim, Germany). The brains were quickly removed and the hypothalamus, hippocampus, amygdala, and prefrontal cortex (PFC) were dissected from both hemispheres and stored at –80°C until mRNA analysis. The spleen was transferred to 0.8% NaCl solution (Fresenius Kabi Deutschland GmbH, Langenhagen, Germany) until isolation of mononuclear cells (see section “Isolation of mononuclear cells from spleens”).

### Hormone and immunoglobulin assays

Milk and plasma cortisol concentrations were measured in duplicate using a commercially available ELISA kit for human samples (DRG Instruments, Marburg, Germany) according to the manufacturer’s instructions. The assay’s sensitivity was 3.4 ng/mL, and the intra- and inter-assay coefficients of variation (CV) were 6.2 and 9.4%, respectively. The assay has been validated for pig cortisol before (Kanitz et al., 2019).

Milk oxytocin was determined using a commercially available ELISA kit (Arbor Assays, Arbor, MI, USA) according to the manufacturer’s instructions. The assay’s sensitivity was 14.8 ng/mL, and the intra- and inter-assay coefficients of variation (CV) were 6 and 7.3%, respectively. Milk supernatants (see above) were diluted 1:10 in assay buffer for the assay.

Plasma concentrations of immunoglobulin A (IgA) were analyzed by porcine-specific enzyme-linked immunosorbent assay (ELISA) according to the manufacturer’s instructions (Bethyl, Laboratories Inc., Montgomery, TX, USA). The assay’s sensitivity was 14.9 ng/mL, and the intra- and inter-assay CVs were <5 and <10%, respectively (Tuchscherer et al., 2012).

### Determination of the neutrophil to lymphocyte ratio from blood

A drop of blood was smeared across a microscopic slide. Blood smears were stained with May-Grünwald solution and subsequently with Giemsa solution (both from Carl Roth, Karlsruhe, Germany) to determine relative leukocyte counts (lymphocytes, monocytes, basophils, eosinophils, neutrophils). At least 200 leukocytes were counted and identified in the smears, and the N/L ratio was calculated.

### Isolation of mononuclear cells from spleens

The collected spleen pieces were transferred into gentleMACS tubes (Miltenyi Biotec, Bergisch-Gladbach, Germany) with 6 ml PBS solution each and disrupted using gentleMACS dissociator (Miltenyi Biotec). Subsequently, the cell suspension obtained was filtered through a cell strainer (70 μm) with 18 ml PBS (phosphate-buffered saline, Sigma-Aldrich/Merck, Darmstadt, Germany), the PBMCs were separated by density gradient centrifugation, and the erythrocytes were lysed [1 ml H_2_O for 15 s, osmolarity reinstalled with 108 μl NaCl solution (8.8 %)]. The cell suspension was then filtered again through a cell strainer (50 μm). The cells were counted using a cell counter (Multisizer™ 3 Coulter Counter, Beckmann Coulter, Krefeld, Germany) and the cell number was adjusted to 2 × 10^6^ cells/ml in culture medium (RPMI-1640, PAN-Biotech, Aidenbach, Germany; 10% FBS, Biochrom, Berlin, Germany; 2 mM glutamine, 50 μg/ml gentamycin, 0.05 mM mercaptoethanol, Sigma-Aldrich/Merck) for the proliferation assay.

### Proliferation assay

Splenic mononuclear cells were seeded at 1 × 10^6^ cells/ml density in culture medium either without (unstimulated control) or with the mitogens concanavalin A (ConA; 25 μg/ml) or lipopolysaccharide (LPS; 12.5 μg/ml; Sigma-Aldrich/Merck) and incubated for 72 h at 37°C and 5% CO_2_ in a 96-well plate (200 μl per well). The plate was centrifuged at 220 × g for 10 min at room temperature and 100 μl of supernatant was taken from each well. Then, 10 μl of MTT (3-[4,5-dimethylthiazol-2-yl]-2,5-diphenyl tetrazolium bromide; Sigma-Aldrich/Merck; 5 mg/ml in PBS) was pipetted into each well. After incubation for 4 h at 37°C and 5% CO_2_, 100 μl of a pre-warmed (37°C) SDS solution (sodium dodecyl sulfate, Sigma-Aldrich/Merck) was added and incubated overnight at 37°C and 5% CO_2_. The metabolic activity of the cells was measured by determining the optical density (550 nm; reference 690 nm) using a micro-plate reader (Spectrostar nano, BMG Labtech, Ortenberg, Germany). The results were expressed as proliferation index (PI), which could be calculated according to the following formula:


(1) $$PI=\frac{OD_{stimulatedcells}}{OD_{unstimulatedcells}}$$

PI values of ≥1.4 were considered proliferation.

### RNA extraction and quantification of transcripts

RNA extraction of brain samples was performed using the RNeasy Lipid Tissue Kit (Qiagen, Hilden, Germany) according to the manufacturer’s protocol. The RNA concentration was determined at 260 nm by using a NanoPhotometer™ (Implen, Munich, Germany) and the purity and integrity were determined by calculating the 260/280 nm ratio. Furthermore, mRNA expression was monitored by reverse transcription (RT) of 750 ng of RNA using the iScript cDNA synthesis kit (Bio-Rad, Munich, Germany) according to the manufacturer’s guidelines. The resulting cDNA was amplified by real-time PCR (iCycler, Bio-Rad) for the following genes: NR3C2 (mineralocorticoid receptor; MR), NR3C1 (glucocorticoid receptor; GR), CRHR1 (corticotropin releasing hormone receptor 1), CRHR2 (corticotropin releasing hormone receptor 2), AVPR1 (arginine vasopressin receptor 1a), OXTR (oxytocin receptor), BDNF (brain-derived neurotrophin receptor), ACTB (actin beta), and TBP (TATA-box binding protein). One microliter of the RT reaction solution was added to 6 μL of iQ SYBR Green Supermix (Bio-Rad) and 4 μL of primer mix with gene-specific oligonucleotides (TIB Molbiol, Berlin, Germany). All the reactions were performed in triplicate. Primers were designed corresponding to the gene sequences of the NCBI database. Whenever possible, primers were designed to span the exon-exon junctions and to anneal between 57^°^ and 61°C. The oligonucleotide sequences of the primers are summarized in Supplementary Table 1.

PCR was performed using a hot start (3 min, 94°C; 30 s, 60°C; 45 s, 70°C), 39 cycles (10 s 94°C; 30 s 60°C; 45 s 70°C with 5 s of time extension per cycle) and a final cycle (10 s 94°C; 30 s 60°C; 7 min 70°C, 1 min 94°C), corresponding to denaturation, annealing, and elongation, respectively. The specificity of the products was assessed using melting point analysis (60°–90°C, 1°C per 10 s), and agarose gel electrophoresis (3.5%). The oligonucleotide structure was verified by sequencing in a subset of the experiments. The relative quantification was calculated using the quantification module of the CFX Manager Software™ version 2.1 (Bio-Rad). Data for mRNA expression of the investigated genes are presented as relative expression ratios normalized to ACTB (beta-actin) and TBP (TATA-box binding protein).

### Statistical analysis

Statistical analysis was performed using SAS software for Windows, version 9.4 (Copyright 2002–2012, SAS Institute Inc., Cary, NC, USA). Descriptive statistics and tests for normality were calculated with the UNIVARIATE procedure of the Base SAS software.

A multifactorial analysis of variance (ANOVA) was used to analyze normally distributed data (suckling interval and milk cortisol) using the SAS procedure MIXED. Fixed effects included trial (levels: 1–10) and treatment (levels: deprivation litter, control litter). Repeated measurements were accounted for by using the statement “repeated” to determine the block diagonal structure of the residual covariance matrix.

Non-normally distributed data were analyzed using the SAS procedure GLIMMIX. Here, the ANOVA model for the analysis of the brain samples included the fixed effects trial (levels: 1–10), treatment (levels: DA, DG, C), sex (levels: male, female), and the interaction “treatment × sex.” The ANOVA model for the blood samples, and OF/NO duration data also included the fixed effect time (levels: PND 16, PND 40) and the interactions “treatment × time” and “treatment × time × sex.” The sow was included as a random effect and measurement replicates on the same animal with the option “random residual,” to determine the block diagonal structure of the residual covariance matrix.

For the analysis of behaviors characterizing the sow-piglet interactions and OF/NO frequency data, a Poisson model was used to determine discrete characteristics. Fixed effects and interactions corresponded to the model above.

Results are presented as least squares means (LSM) and standard errors (SE) for all fixed effects of the above models. Multiple pairwise comparisons of these LS means were performed with the Tukey-Kramer procedure, with the significance level α chosen as 0.05 (test results with *p* ≤α are significant). Results with *p* > 0.05 and ≤0.1 were considered as trends.

## Results

### Mother-offspring relationship

We hypothesized that deprivation would be a psychosocial stressor for both mother and offspring. Therefore, we studied the behavioral and neuroendocrine changes in sows and piglets.

To assess the acute stress response of sows to deprivation treatment, milk samples were collected on the second lactation day before piglet withdrawal and after the return of the piglets and were analyzed for cortisol and oxytocin. There was a significant increase in cortisol in the milk of sows of the deprivation litters compared to sows of the control litters after the first deprivation treatment (Table 1). In contrast, during the deprivation period (lactation days 9 and 15), there was no significant difference in milk cortisol concentrations between sows of deprivation litters and sows of control litters. On lactation day 9, cortisol concentrations were 29.01 ± 2.24 and 32.76 ± 2.28 ng/ml for sows of deprivation and control litters, respectively. On lactation day 15, cortisol concentrations were 31.49 ± 2.24 and 35.39 ± 2.28 ng/ml for sows of the deprivation and control litters, respectively. There was no significant difference between the milk oxytocin of the deprivation and control sows and no significant effect of deprivation itself (Table 1). However, there was a time effect. Oxytocin concentrations on lactation day 9 were 194.96 ± 90.31 and 269.32 ± 85.42 pg/ml, for sows of deprivation and control litters, respectively. They were 223.14 ± 84.61 and 128.62 ± 85.42 pg/ml for sows of deprivation and control litters, respectively, on lactation day 15. There was a significant effect of time (*F*-test; *p* < 0.01) for milk oxytocin concentrations.

**TABLE 1:** Cortisol and oxytocin concentrations in the milk of sows on the second lactation day before and after deprivation treatment.

	Treatment group		*P-values*
Parameter	Deprivation	Control	Treatment
Cortisol (ng/ml)			
Before	22.99 ± 3.05	35.43 ± 2.93	0.077
After	31.19 ± 2.57	26.35 ± 2.92	0.905
Difference (after-before)	7.62 ± 3.70	–7.26 ± 3.70	<0.05
Oxytocin (pg/ml)			
Before	776.38 ± 202.92	944.43 ± 191.08	0.999
After	477.17 ± 166.26	549.14 ± 191.08	1.000
Difference (after-before)	–269.38 ± 452.74	–274.38 ± 452.74	0.994

Results are presented as LSM ± SE of the sows of deprivation and control litters. Tukey-Kramer test; *n* = 7 samples per treatment.

Behavioral interactions between sows and piglets were observed immediately after the reunion of sows and piglets. While maternal behaviors did not show any significant differences between deprivation and control litters, piglets of deprivation litters vocalized more than piglets of control litters. We interpreted the piglet calls as a request for milk because we observed the sows to assume a nursing position (Table 2). The behaviors “pre-lying behavior,” “response to human,” “response to screams,” and “suckling intervals” could not be analyzed because they occurred too infrequently for statistical analysis.

**TABLE 2:** Relative frequencies (%) of behaviors of sows and piglets preceding the first three suckling acts after the end of the deprivation procedure.

	Treatment group		*P-values* (*F*-test)
Behavior	Deprivation	Control	Treatment
Lying down	96.00 ± 22.44	94.48 ± 30.5	0.557
Piglet contact	26.54 ± 3.86	31.33 ± 4.22	0.403
Grunting	44.65 ± 4.84	58.12 ± 5.22	0.083
Piglet calling	59.65 ± 4.79^a^	43.46 ± 5.28^b^	< 0.05

Lying down (sow showed controlled lying down behavior), piglet contact (sow responded to piglets near her head with contact/sniffing), grunting (the sow initiated suckling by calling), piglet calling (piglets initiated suckling by calling). Results are given as LSM ± SE and *p*-values of the *F*-test. Within a row, significant differences are indicated by different letters (^a,b^*p* < 0.05; Tukey-Kramer test; *n* = 231 observations per group).

**FIGURE 1:**
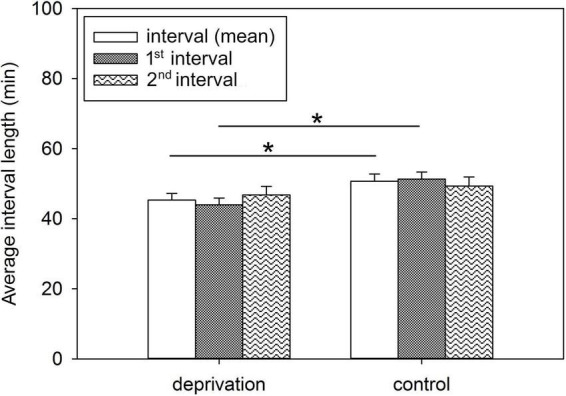
Average interval lengths between suckling bouts of deprivation and control litters. Results are presented as LSM ± SE. Significant differences are marked with asterisks (*p < 0.05; Tukey-Kramer test; *n* = 10 litters/treatment group). 1st interval, interval between 1st and 2nd suckling bout; 2nd interval, interval between 2nd and 3rd suckling bout.

The intervals between the first and second, as well as second and third suckling bouts, were averaged over lactation days 2, 5, 7, 9, 12, 14, and 19 to determine the effect of deprivation treatment on the intervals between suckling bouts after the piglets returned to the sow. Here, statistical analysis revealed a significant treatment effect on the mean interval length [*F*(1, 10) = 5.02; *p* < 0.05] and for the interval length between 1st and 2nd suckling bouts [*F*(1, 10) = 8.93; *p* < 0.05], which were significantly shorter in the deprivation litters than in the control litters (Figure 1). No statistically significant treatment effect was detected for the intervals between 2nd and 3rd suckling bouts [*F*(1, 10) = 0.57; *p* = 0.47]. The suckling intervals did not change significantly over the days. There was a trend toward slightly longer suckling intervals over the days of lactation, while the treatment effect on interval length between the first and second suckling remained as a trend over time (*p* = 0.053). The original data are presented in Supplementary Table 2.

### Effects of maternal deprivation on baseline neuroendocrine and immunological parameters of piglets

Blood samples were collected on PNDs 5 and 16 to assess the effects of maternal deprivation on the baseline stress level of piglets and potential effects on their immune system. There was no significant effect of treatment, sex, or the interaction of treatment × sex on plasma cortisol concentrations, plasma IgA and neutrophil/lymphocyte (N/L) ratio of piglets on PNDs 5 and 16. However, cortisol concentrations tended to be affected by sex on PND 16 (Table 3).

**TABLE 3:** Cortisol concentrations and immunological parameters in the blood of piglets.

	Treatment group			*P-values* (*F*-test)		
Parameter	DA	DG	K	Treatment	Sex	Treatment × sex
**Cortisol (ng/ml)**						
PND 5	33.53 ± 3.21	33.34 ± 3.22	26.46 ± 3.15	0.234	0.725	0.155
PND 16	22.78 ± 3.04	23.09 ± 3.08	17.29 ± 3.01	0.360	0.084	0.265
**IgA (ng/ml)**						
PND 5	2.23 ± 0.32	2.07 ± 0.32	2.02 ± 0.32	0.637	0.695	0.881
PND 16	0.12 ± 0.02	0.10 ± 0.02	0.10 ± 0.02	0.229	0.226	0.451
**N/L ratio**						
PND 5	2.05 ± 0.18	1.97 ± 0.18	2.11 ± 0.18	0.835	0.900	0.925
PND 16	0.82 ± 0.24	0.79 ± 0.24	0.99 ± 0.24	0.818	0.764	0.147

Results are presented as LSM ± SE of the three treatment groups DA (deprivation alone), DG (deprivation in a group with littermates), C (controls, no deprivation), and the *p*-values of the *F*-test. *n* = 50–55 per treatment group for cortisol and IgA; *n* = 40 per treatment group for neutrophil to lymphocyte (N/L) ratio.

Lymphocyte proliferation was tested on spleen mononuclear cells on PND 20. We found no significant treatment effects on the proliferation capacity of spleen cells after ConA or LPS stimulation (Supplementary Table 3). The PI of ConA-stimulated T cells was not significantly affected by “treatment,” “sex,” or the interaction “treatment × sex.” The PI of LPS-stimulated B cells was below the proliferation threshold, indicating that the B cells were not stimulated to proliferate by the dose of LPS used.

### Neuroendocrine and behavioral responses to a challenge

One day after the end of the deprivation period, we exposed the piglets to an open-field/novel-object (OF/NO) test to check how repeated maternal deprivation would affect piglets’ behavioral reactivity to the challenge of brief isolation in an unfamiliar environment. The OF/NO test was repeated on PND 40.

**FIGURE 2:**
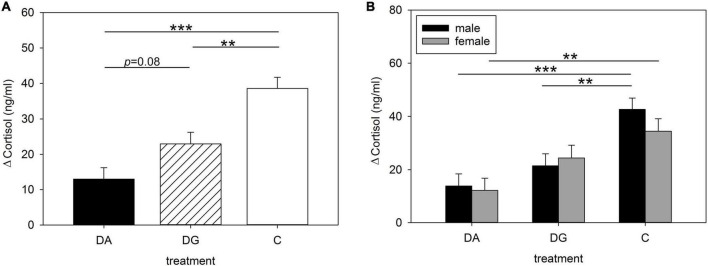
Increases in plasma cortisol concentrations of **(A)** all; **(B)** male and female piglets of the three treatment groups DA (deprivation alone), DG (deprivation in a group with littermates) and C (control, no deprivation) during the open-field/novel-object test. Results are presented as LSM ± SE. Significant differences are marked with asterisks (**p < 0.01; ***p < 0.001; Tukey-Kramer test; *n* = 25–30 per treatment group and sex).

Blood samples from all piglets on PND 16 before and after the OF/NO test were analyzed for cortisol to assess the influence of maternal deprivation on piglets’ cortisol concentrations when exposed to a challenge. We found that the type of deprivation treatment significantly affected the piglets’ cortisol concentrations after the OF/NO test [*F*(2, 133) = 6.02; *p* < 0.01], as well as the difference in these values from before the OF/NO test [*F*(2, 132) = 16.29; *p* < 0.001]. In addition, cortisol concentrations after the OF/NO test were significantly influenced by sex [*F*(1, 133) = 4.54; *p* < 0.05], and in tendency by the interaction of treatment × sex [*F*(1, 132) = 2.92; *p* < 0.056]. Figure 2 shows the results of the pairwise comparisons of treatment. The increase in cortisol by the OF/NO test on PND 16 was greater in C piglets than in DA and DG piglets (Figure 2A). Visualizing male and female piglets separately (Figure 2B) reveals that male C piglets demonstrate stronger increases in cortisol than male DG or DA piglets. Female C piglets have higher increases in cortisol than female DA piglets. In contrast, female DG piglets are not significantly different from female C piglets, indicating that social support has a buffering effect in female DG piglets but not in DG males.

Statistical analysis of OF/NO behavior revealed that the type of treatment had a significant effect on the latency of object contacts [*F*(1, 194) = 4.10; *p* < 0.05] while sex significantly influenced the duration of object contacts [*F*(1, 211) = 3.93; *p* < 0.05]. In addition, day of life significantly influenced the latency [*F*(1, 211) = 17.01; *p* < 0.001], number [*F*(1, 211) = 89.62; *p* < 0.001], and duration [*F*(1, 211) = 22.22; *p* < 0.001] of locomotion, as well as the number of vocalizations [*F*(1, 194) = 98.00; *p* < 0.001] and object contacts [*F*(1, 149) = 12.59; *p* < 0.001]. In addition, relative to age, treatment significantly affected the number [*F*(2, 211) = 6.31; *p* < 0.01] and duration [*F*(2, 211) = 16.08; *p* < 0.001] of locomotion, the latency [*F*(2, 149) = 6.40; *p* < 0.01] and number [*F*(2, 149) = 3.42; *p* < 0.05] of object contacts, and the number [*F*(2, 194) = 15.35; *p* < 0.001] of vocalizations.

**FIGURE 3:**
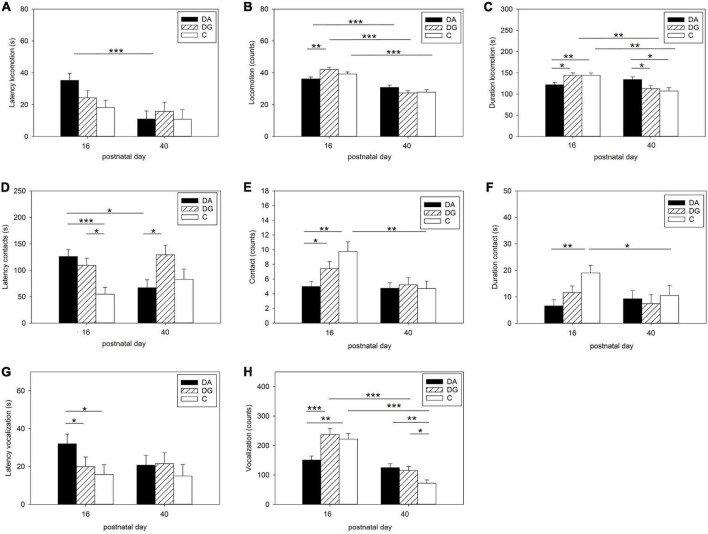
Behavior in the open-field/novel-object test. Latency **(A)**, number **(B)**, and duration **(C)** of locomotion; Latency **(D)**, number **(E)**, and duration **(F)** of contact to the novel object; latency **(G)**, and number **(H)** of vocalizations during OF/NO test in the three treatment groups DA (deprivation alone), DG (deprivation in a group with littermates), and C (control, no deprivation). Results are presented as LSM ± SE. Significant differences (Tukey-Kramer test) are indicated with asterisks (*p < 0.05; **p < 0.01; ***p < 0.001; PND 16: *n* = 52–55; PND 40: *n* = 24–33 per treatment group).

Pairwise comparisons of piglets’ behavior in the OF/NO test showed differences between PND 16 and PND 40 (Figure 3). DA piglets showed a higher latency to locomotion on PND 16 than on PND 40 (Figure 3A). DA piglets showed a shorter duration of locomotion than DG and C piglets on PND 16 (Figure 3C). While all groups moved less often on PND 40 than on PND 16 (Figure 3B) and DG and C piglets also showed a shorter duration of locomotion on PND 40 than on PND 16, DA piglets demonstrated the same duration of locomotion on PND 40 as on PND 16 and for longer than DG and C piglets on PND 40 (Figure 3C).

When confronted with a novel object, the latency to object contact was higher in DA and DG piglets than in C piglets on PND 16. On PND 40, however, DA piglets showed a shorter latency than DG piglets and compared to PND 16 (Figure 3D). The number of contacts was lower in DA piglets than in DG and C piglets on PND 16. C piglets showed significantly fewer contacts to the novel object on PND 40 than on PND 16 (Figure 3E). DA piglets had a shorter duration of contact with the novel object than C piglets on PND 16.

In terms of acoustic signals, DA piglets showed a higher latency to vocalization (Figure 3G) and vocalized less than DG and C piglets (Figure 3H) on PND 16. On PND 40, DA and DG piglets vocalized more than C piglets (Figure 3H). DG and C piglets vocalized less than on PND 16, while this parameter did not change for DA piglets (Figure 3H).

**FIGURE 4:**
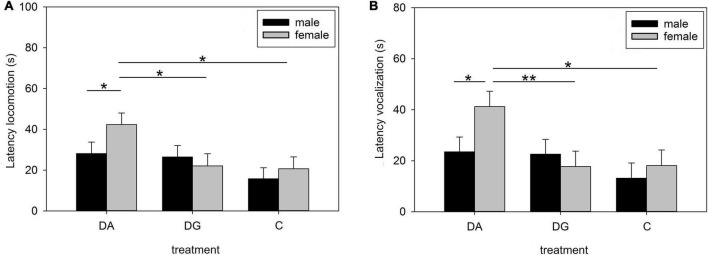
Behavior in the open-field/novel-object test on PND 16. Latency of locomotion **(A)** and vocalization **(B)** of male and female pigs of the three treatment groups DA (deprivation alone), DG (deprivation in a group with littermates), and C (control, no deprivation). Results are presented as LSM ± SE. Significant differences (Tukey-Kramer test) are indicated with asterisks (*p < 0.05; **p < 0.01; *n* = 24–33 per treatment group and sex).

Male and female piglets showed the same OF/NO behavior for all of the above parameters except for latency to locomotion, latency to vocalization, and duration of contact. Female DA piglets had a higher latency to locomotion and vocalization than female DG and C piglets on PND 16 (Figure 4). In fact, they were responsible for the differences between the treatment groups. Male piglets showed a longer duration of object contact than female piglets when all groups were summarized (male: 18.18 ± 1.84 s; females: 8.24 ± 1.97 s; *p* < 0.05).

Interestingly, C piglets showed a shorter duration of contact on PND 40 than on PND 16 (Figure 3F).

### Gene expression in stress-associated brain regions

Psychosocial stress elicits a response from the limbic system and the PFC. Therefore, we studied the expression of genes involved in HPA axis function and regulation in the hypothalamus, amygdala, hippocampus, and PFC.

#### Hypothalamus

**FIGURE 5:**
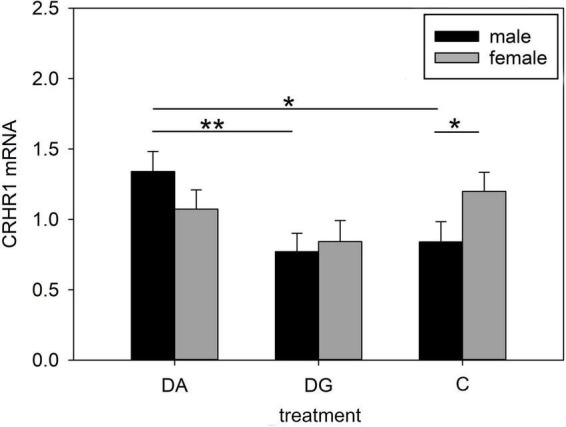
Gene expression in the hypothalamus of piglets on PND 20. CRHR1 (corticotropin releasing hormone receptor 1) mRNA expression of DA (deprivation alone), DG (deprivation with a group of littermates) and C (control, no deprivation) piglets. Data are expressed as arbitrary units after normalization to ACTB and TBP mRNA expression as endogenous reference genes and represent the LS means ± SE. Significant differences are indicated by asterisks (*p < 0.05; **p < 0.01; Tukey-Kramer test; *n* = 10 per treatment group and sex).

Both treatment and the interaction of treatment × sex significantly influenced CRHR1 mRNA expression in the hypothalamus. It was significantly higher in DA piglets than in DG piglets (Table 4). This effect was especially pronounced in male DA piglets, which had a higher CRHR1 mRNA expression than male DG and C piglets. Interestingly, male C piglets had a lower CRHR1 expression than female C piglets. This sex difference was not found in DA and DG piglets (Figure 5).

**TABLE 4:** Relative mRNA expression of HPA-related parameters in the hypothalamus, amygdala, hippocampus and PFC of piglets of the different treatment groups.

	Treatment group			*P-values* (*F*-test)		
Parameter	DA	DG	C	Treatment	Sex	Treatment × sex
**Hypothalamus**						
MR	1.01 ± 0.11	0.90 ± 0.11	0.89 ± 0.11	0.687	0.352	0.487
GR	1.05 ± 0.14	1.10 ± 0.14	1.27 ± 0.14	0.523	0.055	0.087
MR/GR ratio	1.22 ± 0.17	1.02 ± 0.17	0.75 ± 0.17	0.182	0.829	0.284
CRHR1	**1.21 ± 0.11^a^**	**0.81 ± 0.11^b^**	1.02 ± 0.11	**<0.01**	0.567	**<0.05**
CRHR2	1.27 ± 0.14	1.07 ± 0.14	1.11 ± 0.14	0.551	0.495	0.250
AVPR1A	1.21 ± 0.16	1.42 ± 0.16	1.12 ± 0.16	0.437	0.552	0.378
OXTR	0.97 ± 0.21	1.29 ± 0.21	1.27 ± 0.21	0.472	0.697	0.715
**Amygdala**						
MR	1.05 ± 0.15	0.82 ± 0.15	1.24 ± 0.15	0.078	0.297	0.162
GR	0.89 ± 0.10	0.96 ± 0.10	0.85 ± 0.10	0.721	0.866	0.428
MR/GR ratio	1.26 ± 0.35	1.35 ± 0.36	1.78 ± 0.35	0.580	0.070	0.704
CRHR1	1.09 ± 0.13	0.82 ± 0.13	1.16 ± 0.13	0.122	0.645	0.290
CRHR2	1.22 ± 0.18	0.99 ± 0.18	1.20 ± 0.18	0.474	0.568	0.157
AVPR1A	1.11 ± 0.16	1.04 ± 0.16	1.12 ± 0.16	0.882	0.134	0.379
OXTR	1.09 ± 0.11	0.87 ± 0.11	0.79 ± 0.11	0.127	0.428	0.658
**Hippocampus**						
MR	**1.00 ± 0.09^a^**	**0.65 ± 0.09^b^**	0.79 ± 0.09	**<0.01**	0.452	0.248
GR	0.93 ± 0.08	0.99 ± 0.08	0.92 ± 0.08	0.823	0.358	0.293
MR/GR ratio	1.32 ± 0.14	0.93 ± 0.15	0.93 ± 0.14	0.108	0.117	0.304
AVPR1A	1.37 ± 0.28	1.90 ± 0.29	1.69 ± 0.28	0.315	0.566	0.529
OXTR	1.24 ± 0.15	1.31 ± 0.15	1.28 ± 0.15	0.946	0.250	0.596
BDNF	1.13 ± 0.13	1.54 ± 0.13	1.38 ± 0.13	0.100	**<0.05**	0.914
**PFC**						
MR	**0.62 ± 0.05^c^**	**0.64 ± 0.05^c^**	**1.12 ± 0.05^d^**	**<0.001**	**<0.01**	**<0.001**
GR	**0.54 ± 0.04^c^**	**0.54 ± 0.04^c^**	**0.98 ± 0.04^d^**	**<0.001**	**<0.05**	**<0.001**
MR/GR ratio	1.23 ± 0.08	1.29 ± 0.08	1.17 ± 0.08	0.507	0.954	0.083
CRHR1	**0.66 ± 0.05^c^**	**0.66 ± 0.05^c^**	**1.01 ± 0.05^d^**	**<0.001**	0.067	0.067
CRHR2	0.70 ± 0.10	0.67 ± 0.10	1.02 ± 0.10	0.070	0.377	0.724
AVPR1A	1.05 ± 0.14	1.05 ± 0.14	1.31 ± 0.14	0.350	0.738	0.277
OXTR	0.93 ± 0.06	0.85 ± 0.06	1.00 ± 0.06	0.184	**<0.05**	0.212

DA (deprivation alone), DG (deprivation with a group of littermates), and C (control, no deprivation); MR (mineralocorticoid receptor; *NR3C2*), GR (glucocorticoid receptor; NR3C1), CRHR1/CRHR2 (corticotropin releasing hormone receptor 1/2), AVPR1A (arginine vasopressin receptor 1A), OXTR (oxytocin receptor), BDNF (brain-derived neurotrophic factor). Data are presented as LSM ± SE. Within a row, significant differences are indicated by different superscript letters (^a,b^*p* < 0.01; ^c,d^*p*<0.001; Tukey-Kramer test; *n* = 20 pigs/treatment). Bold numbers highlight significant differences.

#### Amygdala

**FIGURE 6:**
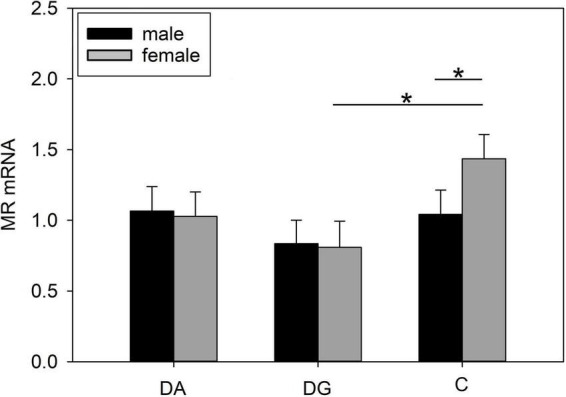
Gene expression in the amygdala of piglets on PND 20. MR (mineralocorticoid receptor; NR3C2) mRNA expression of DA (deprivation alone), DG (deprivation with a group of littermates), and C (control, no deprivation) piglets. Data are expressed as arbitrary units after normalization to ACTB and TBP mRNA expression as endogenous reference genes and represent the LS means ± SE. Significant differences are indicated by asterisks (*p < 0.05; Tukey-Kramer test; *n* = 10 per treatment group and sex).

In the amygdala, we did not find any significant effects between treatment groups (Table 4). However, when analyzing the sexes separately, we found lower MR mRNA expression (Figure 6) in female DG piglets compared to female C piglets, while female C piglets showed a higher MR mRNA expression (Figure 6) than male C piglets.

#### Hippocampus

**FIGURE 7:**
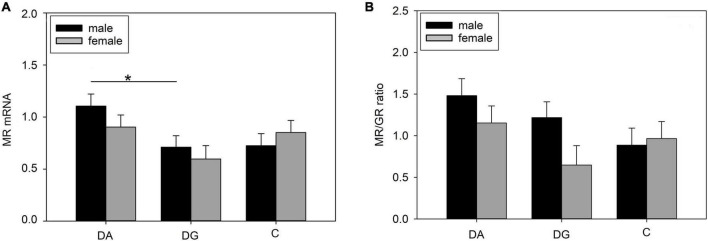
Gene expression in the hippocampus of piglets on PND 20. **(A)** MR (mineralocorticoid receptor; NR3C2) mRNA expression and **(B)** MR/GR ratio of DA (deprivation alone), DG (deprivation with a group of littermates), and C (control, no deprivation) piglets. Data are expressed as arbitrary units after normalization to ACTB and TBP mRNA expression as endogenous reference genes and represent the LS means ± SE. Significant differences are indicated by asterisks (*p < 0.05; Tukey-Kramer test; *n* = 10 per treatment group and sex).

We found a significant treatment effect on MR mRNA expression (Table 4) in the hippocampus. Pairwise comparisons revealed a significantly higher MR mRNA expression in DA than DG piglets. When analyzing both sexes separately, we found this difference was significant for male DA piglets (Figure 7A). The MR/GR ratio tended to be higher in male than in female DG piglets (*p* = 0.07; Figure 7B).

Also, we found a significant sex effect on BDNF mRNA expression (Table 4). It was significantly higher in female than in male piglets (1.51 ± 1.11 vs. 1.19 ± 1.10; *p* < 0.05).

#### Prefrontal cortex

The strongest effects could be observed in the PFC. Treatment had a significant effect on MR, GR, and CRHR1 mRNA expression. All three receptors and in tendency also CRHR2 were more weakly expressed in DA and DG piglets compared to C piglets (Table 4). In addition, there were significant effects of sex and the interaction treatment × sex on MR and GR mRNA expression, while these effects did not reach significance for CRHR1 mRNA expression (Table 4).

**FIGURE 8:**
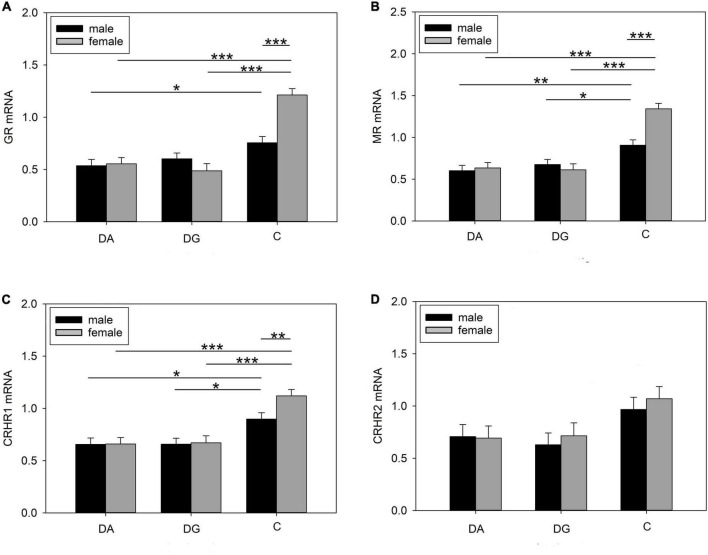
Gene expression in the PFC of piglets on PND 20. **(A)** GR (glucocorticoid receptor; NR3C1), **(B)** MR (mineralocorticoid receptor; NR3C2), **(C)** CRHR1 (corticotropin releasing hormone receptor 1) and **(D)** CRHR2 mRNA expression and MR/GR ratio of DA (deprivation alone), DG (deprivation with a group of littermates), and C (control, no deprivation) piglets. Data are expressed as arbitrary units after normalization to ACTB and TBP mRNA expression as endogenous reference genes and represent the LS means ± SE. Significant differences are indicated by asterisks (*p < 0.05; **p < 0.01; ***p < 0.001; Tukey-Kramer test; *n* = 10 per treatment group and sex).

Both male and female DA piglets showed a lower expression of GR mRNA (Figure 8A), MR mRNA (Figure 8B), and CRHR1 mRNA (Figure 8C) than male and female C piglets, respectively. Female DG piglets had a lower expression of GR mRNA (Figure 8A) than female C piglets. Male and female DG piglets displayed a lower expression of MR (Figure 8B) and CRHR1 (Figure 8C) mRNA than male and female C piglets. Male C piglets expressed less GR, MR, and CRHR1 mRNA than female C piglets (Figures 8A–C). No significant differences have been found for CRHR2 (Figure 8D).

The OXTR mRNA expression showed a significant sex effect (Table 4). Pairwise comparisons revealed a higher OXTR expression in female than in male C piglets (female: 1.15 ± 0.079; male: 0.855 ± 0.079; *p* < 0.01).

## Discussion

### Mother-offspring interaction

This study addressed the consequences of repeated maternal deprivation of piglets during lactation with and without a group of conspecifics. Analysis of cortisol in milk showed an increase during the deprivation procedure in sows of the deprivation litters. One explanation for the tendency of higher cortisol levels on the second day of lactation in the control group compared to the deprivation group before the start of deprivation treatment could be the increase in cortisol during parturition. The experiment started at the same time for all sows, but the interval to farrowing varied individually by several hours. Sows were randomly assigned to the deprivation or control group on the second day of lactation. Therefore, the differences between the two groups of sows before treatment were due to random individual differences. Nevertheless, to compare the groups, we calculated the difference in cortisol levels between the end and the beginning of the first deprivation treatment on the second day of lactation. While cortisol levels should naturally decrease during the course of the deprivation treatment as observed in the control sows, the deprivation sows showed an increase in cortisol levels due to stress.

Maternal behavior such as controlled laying down to avoid piglet crushing and responses to piglets near the sow’s head by touching or sniffing showed no significant differences between mothers of deprivation and control litters. This was a bit surprising, as studies in rats have shown increased maternal behavior such as licking and grooming after the pups were returned from an isolation treatment (Kosten and Kehoe, 2010). On the other hand, comparing the maternal behavior of wild-type sows (a cross between wild boar and domestic pigs) and domestic sows revealed less nose contact between the domestic sows and their piglets (Gustafsson et al., 1999). Therefore, it could be assumed that this maternal behavior is less pronounced in domesticated sows than in their wild ancestors and should not be overestimated when evaluating sow-piglet interactions. A recent study compared maternal behavior directly after farrowing in modern-type sows and old-type sows obtained by inseminating modern French Large White with frozen semen of boars born in 1977–1998 (Canario et al., 2014). While the study shows a higher maternal responsiveness of modern-type sows toward their newborn piglets in the second parity, the probability of nose contact decreases with time within the observational frame of 6 h after farrowing. However, the study demonstrates that genetic components have a strong influence on maternal behavior. Nevertheless, there were differences in sow-offspring interactions. The piglets of the deprivation litters vocalized significantly more than the piglets of the control litters to initiate suckling by calling their mother. This vocalization could indicate hunger, as the piglets had missed meals due to deprivation and voiced their urgent need for food. This is evident from the suckling intervals immediately after the return of maternally deprived piglets. The interval between the first and the second suckling bout was shorter in deprived than in control litters, whereas the interval between the second and the third suckling bout was not. It may also have been a way for them to express their need for comfort from their mothers. In turn, the sows of the deprivation litters tended to call their piglets less to initiate suckling. Perhaps they did not have to call for very long because the piglets were eager to suckle. On the other hand, it may reflect the same stress-induced phenomenon as observed in a study of the effects of prenatal maternal social stress on maternal behavior. Sows that were exposed to social mixing stress during gestation, showed increased latencies to respond vocally to an isolated piglet’s call compared to control sows (Ringgenberg et al., 2012). This indicates that stress may affect maternal behavior.

Contrary to our expectations, we did not find more effects of deprivation on maternal behavior. Of note, these sows were in the second to fourth parity. Therefore, they had already experienced husbandry-related separations from their very young offspring and could have become habituated to such stressors. In addition, lactation has a dampening effect on the activity of the HPA axis (Altemus et al., 1995; Cook, 1997; Brunton et al., 2008; Windle et al., 2013). As explained above, measurement of cortisol levels in milk on the second day of lactation may have been biased by the short time interval from farrowing when cortisol is elevated (Lawrence et al., 1997; Jarvis et al., 1998). Nevertheless, sows of deprivation litters showed a stronger increase in milk cortisol than control sows, which may indicate that they had been stressed.

### Cortisol and immune parameters of piglets

Analysis of piglets’ blood samples on PNDs 5 and 9 revealed no deprivation-induced effects, neither on cortisol nor on IgA concentrations and N/L ratios. A previous article showed increased plasma IgA after stress in pigs (Kanitz et al., 2019). Human studies on stress often use secretory IgA (sIgA) as a non-invasive marker for stress (Tsujita and Morimoto, 1999). Secretory IgA plays an important role in defense against infections on mucosal surfaces. It is often found at elevated levels immediately after stress and at reduced levels later on (Tsujita and Morimoto, 1999). While sIgA is usually produced by plasma cells in the mucosa (Brandtzaeg, 2013), it has been shown in pigs that plasma IgA corresponds well to intestinal sIgA (Vaerman et al., 1997).

The N/L ratio reflects glucocorticoid-mediated effects on the immune system and is therefore often used as a proxy for stress-induced immunomodulation because cortisol increases neutrophil proliferation and reduces lymphocyte proliferation (Davis et al., 2008). Also, the proliferative abilities of spleen lymphocytes in response to mitogens were not affected. This indicates that a repeated stressor does not necessarily result in immunomodulation when no additional challenge exists. However, a challenge such as the OF/NO test induced a differential effect on the cortisol concentrations of the three treatment groups (see below). Moreover, we were able to show in a related study (Brückmann et al., 2020) that the piglets of the three treatment groups reacted differently to an immunological challenge such as intraperitoneal injection of lipopolysaccharide, mimicking a bacterial infection, as late as PND 42. In the study concerned, DA and DG piglets showed stronger sickness behavior and weaker immune responses than C piglets after LPS administration (Brückmann et al., 2020).

### Open-field/novel-object behavior of piglets

The behavior of DA piglets in the OF/NO test on PND 16 could be interpreted as less aroused than that of DG and C piglets. They had a higher latency to vocalization and they vocalized less, which was attributed by Puppe et al. (2007) to lower arousal. This could be due to the previous deprivation treatment of these piglets. They were isolated a total of 14 times until 1 day before the test, allowing them to become habituated to being alone.

This habituation to solitude can be compared to the situation when an OF/NO test was repeated the next day (Donald et al., 2011) or after 4 h (Kanitz et al., 2009), in which piglets vocalized less and showed less active behavior, which is in line with our finding of shorter duration of locomotion in DA piglets compared to DG and C piglets. On the other hand, both DA and DG piglets showed a higher latency to contact with the new object. In addition, DA piglets exhibited fewer contacts with the new object than DG and C piglets did, as well as a longer contact duration than C piglets on PND 16. According to Puppe et al. (2007), this could be interpreted as more fearful behavior in DA piglets than in DG and C ones. In contrast, DA piglets showed the lowest increase in cortisol during the OF/NO test. This apparent contradiction is clarified when we consider that the lower cortisol increase in DA piglets may indicate a negative feedback regulation of cortisol release due to the previous repeated isolation instead of a lower perceived stress level.

When comparing the first and the second OF/NO test, all piglets showed reduced locomotion counts in the OF/NO test on PND 40, but only DG and C piglets displayed a shorter duration of locomotion in the second OF test. At this time, DA piglets exhibited a significantly longer duration of locomotion than DG and C piglets and no difference from their own behavior on PND 16. This is rather unusual as repeated OF/NO tests have been shown to reduce locomotion (Désautés et al., 1997; Rutherford et al., 2006; Kanitz et al., 2009; Donald et al., 2011). While DG and C piglets vocalized significantly less in the second OF/NO test on PND 40, DA piglets vocalized as much as on PND 16, which can be interpreted as higher arousal of DA piglets. Similar behavior was observed in piglets subjected to an OF/NO test immediately before and after a single 4-h isolation (Kanitz et al., 2009).

Behavioral differences between DA and DG piglets suggest that social support from a group of familiar conspecifics can compensate for maternal deprivation. Even the presence of a single conspecific during isolation without any physical interaction has been shown to blunt the behavioral responses of isolated piglets (Kanitz et al., 2014).

### Gene expression in different brain regions

Expression of HPA-associated genes in stress-related brain areas was affected by deprivation treatment. CRHR1 mRNA expression in the hypothalamus was higher in DA than in DG piglets. Male rats, exposed to prenatal hypoxia stress, had a higher CRHR1 mRNA expression in the paraventricular nucleus of the hypothalamus than male control rats and showed higher anxiety. In contrast, females had a reduced CRHR1 mRNA expression and did not show changes in anxiety-like behavior. This suggests local positive feedback of CRH production of hypothalamic neurons, which may lead to increased anxiety (Wang et al., 2013). Conditional knock-out mice, which did not express CRHR1 in the forebrain, including limbic areas were exposed to early-life stress in the form of limited nesting and bedding material (Rice et al., 2008) and showed reduced anxiety and increased exploration in the elevated-plus maze and the light-dark box (Wang et al., 2012). Conversely, increased expression of CRHR1 in the hypothalamus could explain the behavioral characteristics of DA piglets that indicate increased anxiety, such as higher latency to contact and a shorter duration of contact with the new object on PND 16. DG piglets did not differ significantly from C piglets in CRHR1 mRNA expression in the hypothalamus, which indicates that they were at least partly protected from the stress of maternal deprivation by social support of their littermates.

In the hippocampus, MR mRNA expression was higher in DA than in DG piglets. This is surprising as a number of studies have shown that chronic stress and depressive behavior are correlated with decreased MR mRNA expression in the hippocampus, and decreased MR expression is generally linked to increased basal and stress-induced HPA axis activity (Vázquez et al., 1996; De Kloet et al., 1998; Arabadzisz et al., 2010; Kanatsou et al., 2015). Furthermore, in pigs, a single 4-h isolation decreased MR mRNA expression in the hippocampus of piglets (Kanitz et al., 2014). However, mice with a high hippocampal MR expression displayed reduced anxiety and a diminished HPA-axis activation during stress (De Kloet et al., 2016). Increased MR mRNA expression in DA piglets in this study was accompanied by a diminished cortisol response in the OF/NO test but by more fearful behavior of DA than DG and C piglets. The MR mRNA expression of DG piglets in the hippocampus was similar to that of C piglets, again indicating social buffering from stress effects by the presence of littermates.

Mizoguchi et al. (2008) found increased GR expressions in the hippocampus of chronically stressed rats, resulting in attenuated negative feedback from glucocorticoids. Kanitz et al. (2004) demonstrated significantly increased GR binding in the hippocampus in their study of repeatedly maternally deprived pigs. However, this effect was not apparent until 45 days after cessation of deprivation treatment and was not present in analyses immediately after maternal deprivation. This result is consistent with those of this study, where GR mRNA expression was not altered in the hippocampus on PND 20.

BDNF is a neurotrophin that contributes to synaptic plasticity and neurite outgrowth and is important in controlling learning behavior and memories. It is co-expressed with GR and MR in hippocampal neurons. The cross-talk between glucocorticoids and BDNF shapes HPA-axis development in early life. During this period, high BDNF and low glucocorticoids are necessary for neurons. There is plenty of evidence that early-life adversity shifts the BDNF-glucocorticoid balance and may cause long-term stress vulnerability (Daskalakis et al., 2015). Also, BDNF is inhibited by chronic stress (Suri and Vaidya, 2013). Conversely, elevated BDNF levels may attenuate the negative effects of stress exposure and protect against associated affective disorders (Taliaz et al., 2011). However, no effect of treatment on the expression of *BDNF* in the hippocampus was detected in this present study.

In the PFC, both DA and DG piglets showed lower expression of *MR*, *GR*, and *CRHR1* than C piglets. The PFC plays a central role in processing information and assessing emotional states (Amat et al., 2005; Radley et al., 2006). In addition, it has an important function in regulating the HPA axis (Sullivan and Gratton, 1999, 2002; Dedovic et al., 2009). For example, chronic stress resulted in the decreased expression of GR, MR, and CRHR1 in the PFC of rodents and monkeys (Chen et al., 2008; Patel et al., 2008; Ga̧dek-Michalska et al., 2013). Mizoguchi et al. (2008) found that GR expressions were decreased in the PFC of chronically stressed rats along with reduced negative feedback from glucocorticoids.

AVP and OXT have been shown to influence HPA activity (Engelmann et al., 2004). Their receptors AVPR1a and OXTR are expressed in a wide range of brain regions, including the hypothalamus, amygdala, hippocampus, and PFC (Dumais and Veenema, 2016; Brydges et al., 2020). Maternal separation in rodents altered AVPR1a and OXTR function in the limbic system (Veenema, 2012). However, we did not find any stress-induced changes in AVPR1a or OXTR mRNA expression. Schmidt et al. (2018) showed that stress in mice during pregnancy changed this system in a way that the maternal behavior of stressed dams correlated with hippocampal AVPR1a and OXTR expression in female foster pups that were not stressed. Thus, their AVPR1a and OXTR expression was solely influenced by maternal behavior. In our study, maternal behavior after the deprivation procedure was unchanged. This is possibly the reason for the unchanged *AVPR1a* and *OXTR* expression.

### Sex differences

The behavior of female piglets in the OF/NO test differed from that of male piglets in the parameters “latency to locomotion” and “latency to vocalization” on PND 16, indicating lower arousal than in male piglets. Although DA piglets of both sexes showed low cortisol responses to this OF/NO test, only male DA piglets showed an upregulated hypothalamic CRHR1 mRNA expression compared to male DG and C piglets and a hippocampal MR mRNA expression that was higher than in male DG piglets. Increased hypothalamic CRHR1 mRNA expression was also found exclusively in male rats after prenatal stress, which was attributed to differential epigenetic modifications and accompanied by higher anxiety (Wang et al., 2013). Sex differences in MR function in rats in response to stress have also been described by De Kloet et al. (2016).

With regard to the C piglets, several genes were higher expressed in females than in males. These were *CRHR1* in the hypothalamus; *MR* in the amygdala, and *MR*, *GR*, *CRHR1*, and *OXTR* in the PFC. Interestingly, these sex differences disappeared under stress and were no longer present in DA and DG piglets. Therefore, it could be hypothesized that these sex differences in the expression of stress-related genes were responsible for the differences in OF/NO behavioral “latency to locomotion” and “latency to vocalization” observed mainly in female DA pigs. Apparently, the behavioral reactivity of female DG pigs was buffered by social support during maternal separation. In relation to OXTR, it was observed that OXTR binding was higher in female than in male voles (Smeltzer et al., 2006).

Furthermore, *BDNF* expression in the hippocampus was generally marginally higher in females than in males, but this difference only became significant when all three treatment groups were analyzed together. This is in line with a study in rats, in which females had higher BDNF concentrations (Chan and Ye, 2017). These findings suggest that females have higher neuroplasticity and may be better protected against the negative consequences of early-life stress on HPA-axis regulation and affective behavior. With respect to the stress response, this is consistent with the present study, in which male piglets showed a stronger cortisol response and female piglets were less aroused than males in the OF/NO test, as inferred from their behavior as described above. Sex-specific differences in HPA-axis responses to psychological stress have also been observed in humans, where males had higher cortisol responses before and after puberty (Kudielka and Kirschbaum, 2005).

## Conclusion

Early-life adversity by repeated maternal deprivation of domestic piglets stresses both sows and piglets. While maternal behavior is not affected, piglets show strong and sustained alterations in OF/NO behavior and changes in gene expression in limbic areas and the PFC, suggesting an altered stress regulation system. The baseline immune parameters of the piglets were not affected, but the possible occurrence of stress-induced immunomodulation may be better assessed during a real immunological challenge. Social support effects, as well as sex-specific stress effects, could be seen in OF/NO behavior and gene expression in the brain.

## Data availability statement

The original contributions presented in this study are included in the article/Supplementary material, further inquiries can be directed to the corresponding author.

## Ethics statement

The animal study was reviewed and approved by Landesamt für Landwirtschaft, Lebensmittelsicherheit und Fischerei, Mecklenburg-Vorpommern, Germany; LALLF M-V/TSD/7221.3-1.1-003/18.

## Author contributions

EK and MT contributed to the conception and design of the study. MT, EK, and RB performed the experiments and collected and analyzed the data. AT performed the statistical analyses. UG, RB, and EK wrote the manuscript. All authors interpreted the data.
