# Supplementary material for: Early-life maternal deprivation affects the mother-offspring relationship in domestic pigs, as well as the neuroendocrine development and coping behavior of piglets
Source: Front Behav Neurosci. 2022 Oct 6;16:980350. doi: 10.3389/fnbeh.2022.980350 (PMC9582528; doi:10.3389/fnbeh.2022.980350)
Supplement: Supplementary file 1 [file Table_1.DOCX]

*Supplementary Table 1: Primer sequences, amplicon sizes and reference numbers of gene banks*

| **Gene** | **GeneBank Accession Numbers** | **Sense-, Antisense-Primer (5’-3’)** | **Amplicon (bp)** |
| --- | --- | --- | --- |
| MR | ENSSSCG00000037766 | AGTGTTCTTCAAAAGAGCAGTGG, CCTCGTGGATCCCTTTCAAC | 188 |
| GR | NM_001008481.1 | GTTCCAGAGAACCCCAAGAGTTCA, TCAAAGGTGCTTTGGTCTGTGGTA | 173 |
| CRHR1 | NM_001144110.1 | CTCATCTCAGCCTTCATCCTG, CGAACATCCAGAAGAAGTTGG | 151 |
| CRHR2 | NM_001144118.1 | CAGGGTTTCTTCGTGTCTGTC, GTCTGCTTGATGCTGTGGAAG | 173 |
| AVPR1A | ENSSSCG00000033383.2 | GACCTTCGTGATCGTAACAGC, TCAAGGAAGCCAATAATGCAG | 143 |
| OXTR | NM_214027.1 | GCCTTCATCGTATGCTGGAC, TCTTCAGGTGGCTGGAAGAG | 214 |
| BDNF | NM_214259.2 | GACGAGGACCAGAAAGTTCG, CCTCATGGACATGTTTGCAG | 162 |
| ACTB* | ENSSSCT00000042531 | TCTGGCACCACACCTTCT, TGATCTGGGTCATCTTCTCAC | 114 |
| TBP* | NM_003194.5 | AACAGTTCAGTAGTTATGAGCCAGA, AGATGTTCTCAAACGCTTCG | 153 |

MR (mineralocorticoid receptor; NR3C2), GR (glucocorticoid receptor; NR3C1), CRHR1/CRHR2 (corticotropin releasing hormone receptor 1/2), AVPR1A (arginine vasopressin receptor 1A), OXTR (oxytocin receptor), BDNF (brain-derived neurotrophic factor), ACTB (actin beta), TBP (TATA-box binding protein); * reference genes
