# Supplementary material for: Early-life maternal deprivation affects the mother-offspring relationship in domestic pigs, as well as the neuroendocrine development and coping behavior of piglets
Source: Front Behav Neurosci. 2022 Oct 6;16:980350. doi: 10.3389/fnbeh.2022.980350 (PMC9582528; doi:10.3389/fnbeh.2022.980350)
Supplement: Supplementary file 2 [file Table_2.DOCX]

Supplementary Table 2: Interval lengths between suckling bouts of deprivation and control litters

|  | **Treatment group** | | ***p*-values (F-test)** | | |
| --- | --- | --- | --- | --- | --- |
| **Parameter** | **Deprivation** | **Control** | **Treatment** | **Time** | **Treatment** × **Time** |
| *Average interval (min)* | |  | 0.069 | 0.073 | 0.265 |
| LacDay 2 | **39.25 ± 3.95^c^** | **56.31 ± 4.02^d^** |  |  |  |
| LacDay 5 | 44.20 ± 3.95 | 43.67 ± 4.02 |  |  |  |
| LacDay 7 | 41.61 ± 3.95 | 48.94 ± 4.02 |  |  |  |
| LacDay 9 | 43.88 ± 3.95 | 51.08 ± 4.02 |  |  |  |
| LacDay 12 | 46.29 ± 3.95 | 47.94 ± 4.02 |  |  |  |
| LacDay 14 | 46.70 ± 3.95 | 51.13 ± 4.02 |  |  |  |
| LacDay 19 | 55.34 ± 3.95 | 55.81 ± 4.02 |  |  |  |
| *Interval between 1^st^ and 2^nd^ suckling bout (min)* | |  | **<0.05** | 0.078 | 0.053 |
| LacDay 2 | **37.26 ± 4.50^e^** | **60.23 ± 4.55^f^** |  |  |  |
| LacDay 5 | 44.08 ± 4.50 | 42.60 ± 4.55 |  |  |  |
| LacDay 7 | 46.26 ± 4.50 | 50.14 ± 4.55 |  |  |  |
| LacDay 9 | 41.26 ± 4.50 | 51.42 ± 4.55 |  |  |  |
| LacDay 12 | 45.08 ± 4.50 | 46.23 ± 4.55 |  |  |  |
| LacDay 14 | **37.17 ± 4.50^a^** | **52.14 ± 4.55^b^** |  |  |  |
| LacDay 19 | 56.63 ± 4.50 | 56.33 ± 4.55 |  |  |  |
| *Interval between 2^nd^ and 3^rd^ suckling bout (min)* | |  | 0.477 | 0.109 | 0.788 |
| LacDay 2 | 42.98 ± 5.71 | 45.23 ± 5.73 |  |  |  |
| LacDay 5 | 44.15 ± 5.14 | 45.04 ± 5.22 |  |  |  |
| LacDay 7 | 36.78 ± 5.14 | 48.04 ± 5.22 |  |  |  |
| LacDay 9 | 46.33 ± 5.14 | 51.04 ± 5.22 |  |  |  |
| LacDay 12 | 47.33 ± 5.14 | 49.95 ± 5.22 |  |  |  |
| LacDay 14 | 56.06 ± 5.14 | 50.40 ± 5.22 |  |  |  |
| LacDay 19 | 53.88 ± 5.14 | 55.58 ± 5.22 |  |  |  |

Three suckling bouts after the return of the deprived piglets to their mothers have been analysed. Data are presented as LSM ± SE of piglets of control and deprivation litters on selected lactation days (LacDay). Within a row, significant differences are indicated by different superscript letters (^a,b^ *p* < 0.05; ^c,d^ *p* < 0.01; ^e,f^ *p* < 0.001; Tukey-Kramer test; n = 10 litters/ treatment group).
