# Supplementary material for: Early-life maternal deprivation affects the mother-offspring relationship in domestic pigs, as well as the neuroendocrine development and coping behavior of piglets
Source: Front Behav Neurosci. 2022 Oct 6;16:980350. doi: 10.3389/fnbeh.2022.980350 (PMC9582528; doi:10.3389/fnbeh.2022.980350)
Supplement: Supplementary file 3 [file Table_3.DOCX]

*Supplementary Table 3: Proliferation indices (PI) of splenocytes in response to the T-cell mitogen ConA and the B-cell mitogen LPS*

|  | **Treatment group** | | | ***p*-values (F-test)** | | |
| --- | --- | --- | --- | --- | --- | --- |
| **Proliferation index** | **DA** | **DG** | **C** | **Treatment** | **Sex** | **Treatment** × **Sex** |
| ConA | 2.87 ± 0.57 | 2.69 ± 0.57 | 3.39 ± 0.57 | 0.687 | 0.293 | 0.328 |
| LPS | 1.12 ± 0.10 | 1.25 ± 0.10 | 1.39 ± 0.10 | 0.062 | 0.215 | 0.305 |

Results are presented as LSM ± SE of the three treatment groups DA (deprivation alone), DG (deprivation in a group with littermates), C (controls, no deprivation), and the p-values of the F-test (n = 20 piglets/treatment).
